# Assessing the Relationship Between the Eichner Index and Articular Eminence Inclination in Temporomandibular Dysfunction: A Cross‐Sectional Study

**DOI:** 10.1002/cre2.70286

**Published:** 2026-01-09

**Authors:** Maryam Paknahad, Leila Haghighi, Sonia Dokohaki

**Affiliations:** ^1^ Oral and Dental Disease Research Center, Department of Oral and Maxillofacial Radiology, School of Dentistry Shiraz University of Medical Sciences Shiraz Iran; ^2^ Student Research Committee, Depatment of Pediatric Dentistry, School of Dentistry Shiraz University of Medical Sciences Shiraz Iran; ^3^ Oral and Maxillofacial Radiology, Department of Oral and Maxillofacial Radiology, School of Dentistry Iran University of Medical Sciences Tehran Iran

**Keywords:** articular eminence inclination, cone beam computed tomography, Eichner Index, temporomandibular joint

## Abstract

**Background:**

The articular eminence is essential for the proper functioning of the temporomandibular joint (TMJ), guiding the movement of the mandibular condyle along its posterior slope. Changes in the inclination of the articular eminence (AEI) can occur as a result of tooth loss or aging. The objective of this study was to investigate the association between the AEI and the Eichner Index.

**Materials and Methods:**

The study analyzed 162 cone beam computed tomography (CBCT) images from patients with TMJ dysfunction. These images were categorized into three groups (A, B, and C) according to the Eichner Index. The posterior surface of the AEI was measured using the top‐roof line method. An ANOVA test was conducted to assess the relationship between the Eichner Index and AEI.

**Results:**

A statistically significant relationship has been found between the AEI and the Eichner Index (*p* = 0.005). Class C had a significantly lower inclination than Classes A and B.

**Conclusion:**

This study revealed that tooth loss impacts the AEI, with patients who have more missing teeth exhibiting a significantly flatter AEI. To prevent morphological changes and potential future issues in the TMJ, it is essential to ensure proper dental care and maintain occlusal support for these patients.

## 1 Introduction

The temporomandibular joint (TMJ) is a complex and versatile joint that connects the skull to the mandible, enabling a range of activities such as eating, chewing, swallowing, speaking, and making facial expressions (Vîrlan et al. 2021). The articular eminence is a bony structure that is located in front of the glenoid fossa and behind the zygomatic bone (Bag 2014). The articular eminence inclination (AEI) is the angle formed by the posterior wall of the articular eminence and a horizontal reference plane, such as the Frankfurt plane (Kranjčić et al. 2016). The normal value of this angle in adults ranges from 30 to 60 degrees. Angles below 30 degrees are considered flat, and values above 60 degrees are regarded as steep (Ozkan et al. 2012).

Multiple factors can contribute to changes in the components of the TMJ, including age‐related dental conditions (like tooth loss, wear, or increased functional demands), degenerative changes in the condylar head and glenoid fossa, chewing stress, and the development of the skull and facial structures (Kranjčić et al. 2016). Changes in the shape of the TMJ, such as the flattening of the articular eminence, are more likely to result from tooth loss or reduced occlusal support than from aging (Vîrlan et al. 2021; Csadó et al. 2012; Oruba et al. 2020). Tooth loss can alter the morphology of the TMJ in various ways. The absence of posterior teeth disrupts the balance between adaptation and functional harmony, leading to a loss of the occlusal curve, which may result in decreased functionality (Shehab et al. 2011). Moreover, the inclination of the articular eminence may be affected by the loss of teeth through the remodeling process.

The Eichner Index, originally established by Karl Eichner, is a standard classification system widely utilized in dental epidemiology to assess occlusal stability. This index evaluates functional dental status by identifying intermaxillary contacts within the posterior region, dividing the dentition into four distinct support zones based on the interaction between opposing premolars and molars (Paknahad et al. 2023; Hiltunen et al. 2002).

Various methods have been used to evaluate the articular eminence, such as dry skull measurements, conventional imaging, magnetic resonance imaging (MRI), and computed tomography (CT). These methods have certain limitations. Recently, cone beam computed tomography (CBCT) has become more widely used than CT. It allows for the measurement of angles and distances with accurate dimensions, without overlapping structures, and is significantly more economical than CT in terms of radiation dose and cost (Hintze et al. 2007).

Some studies have examined the relationship between edentulism and the AEI (Csadó et al. 2012; Oruba et al. 2020; Chiang et al. 2015; Jasinevicius et al. 2006a; Jyothi et al. 2020; Modgi et al. 2020; Suhail et al. 2018; Zabarović et al. 2000; Unal Erzurumlu and Celenk 2020; Arıkan et al. 2022; Verma et al. 2020; Indriastuti et al. 2019; Fang et al. 2020; Bichir et al. 2020; Chairunnisa and Harahap 2022; Vîrlan et al. 2024; Anbiaee et al. 2024; Jasinevicius et al. 2006b). These studies have shown inconsistent results on the effect of tooth loss on the AEI. Because of this inconsistency, and given the Eichner Index's advantage of providing a simple functional assessment based on posterior intermaxillary contacts, the present study aimed to examine the association between the Eichner Index and the AEI in patients with TMD using CBCT.

## 2 Materials and Methods

This retrospective cross‐sectional study was approved by the Institutional Ethics Committee (approval code: IR.SUM.DENTAL.REC.1401.088). CBCT images of 162 patients exhibiting clinical signs and symptoms of TMD, including clicking sounds, mouth opening deviation, restricted mouth opening, and tenderness around the TMJ and masticatory muscles, were retrieved from the archives of the oral and maxillofacial radiology department at Dental Faculty of Shiraz University of Medical Sciences. The exclusion criteria were patients with a history of temporomandibular surgery, acute trauma, congenital anomalies, musculoskeletal or neurological diseases, and any systemic disease that could affect joint morphology.

### 2.1 Imaging Procedure

A NewTom VGi (NewTom, Verona, Italy) was used to obtain CBCT images of the TMJ at a voltage of 110 kV, a current of 3.05 mA, and an exposure time of 3.6 s in standard resolution mode (0.3 voxel size). The field of view was 15 × 15 cm. To control for variability in head posture, the patient's position was standardized using the device's cephalostat, which included a chin rest and lateral head supports. Laser alignment beams were utilized to orient the patient's mid‐sagittal plane perpendicular to the floor and the Frankfurt horizontal plane parallel to the floor. To standardize mandibular position, all images were acquired with the teeth in maximum intercuspation, and patients were instructed to avoid swallowing or movement during the scan.

### 2.2 AEI Measurement

**Figure 1:**
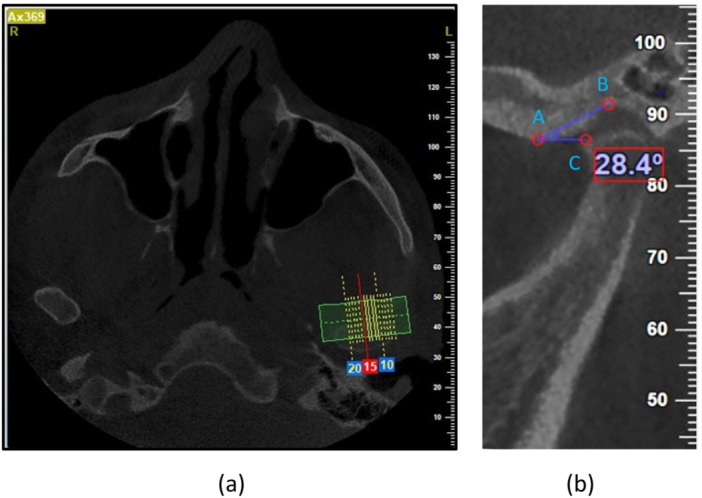
Lowest point of the crest of the AE (A), the highest point of the roof of the glenoid fossa (B), and the Frankfurt plane (C). (a) Axial view and (b) corrected sagittal view.

The AEI was measured using the top‐roof line method (Figure 1). The angle between the Frankfurt plane and the plane passing through the highest point of the roof of the glenoid fossa and the lowest point on the crest of the articular eminence was measured in the sagittal plane.

### 2.3 Tooth Loss Recordings

The patients were classified into three categories (A, B, and C) based on the Eichner Index. In Eichner's Class A, all four occlusal areas are in contact with each other. In Class B, at least one occlusal region is absent, or all four occlusal regions are absent, but the anterior region remains intact. In Class C, there is no occlusal contact between the remaining teeth. This division considers both fully or partially erupted permanent teeth, extra teeth, third molars, pontics, and implants. A dentistry student and an oral and maxillofacial radiologist analyzed the AEI and Eichner Index for every CBCT image. Fifty images were randomly chosen and analyzed 2 weeks later by the student. The collected data were then analyzed using the intraclass correlation coefficient (ICC) for comparison.

### 2.4 Statistical Analyses

Statistical analysis was performed using SPSS (version 22, Chicago, Illinois, the United States). A *t*‐test was used to examine the relationship between gender and the AEI. ANOVA was employed to assess the relationship between the Eichner Index (A, B, and C) and AEI. Additionally, a *t*‐test was used to compare AEI between the two age groups (above 45 years and under 45 years).

## 3 Results

**Figure 2:**
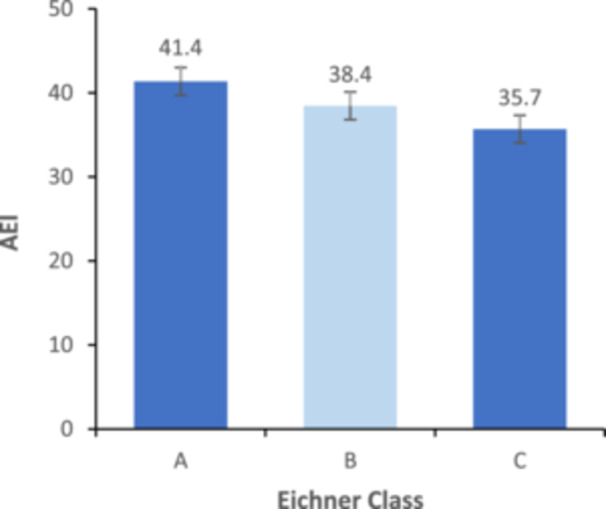
Comparison of articular eminence inclination in different Eichner groups.

This study included 162 patients, of whom 108 were women aged between 18 and 75 years, with a mean of 43.35 (SD 15.27), and 54 men aged between 17 and 72 years, with a mean of 63.63 (SD 27.15). The ICC value of 0.958 demonstrates a high level of consistency between the initial and repeated measurements, confirming the reliability of the data. Additionally, a significant level of agreement was observed among all operators for each measurement (ICC ≥ 0.90). According to the Eichner Index, the order of frequency of the groups was as follows: Group A (58%), Group B (25.9%), and Group C (16%). The AEI was not significantly different between male and female (*p* = 0.235). Table 1 shows a significant relationship between AEI and Eichner Index. The AEI was significantly lower in Class C than in Classes A and B (Figure 2). Table 2 demonstrates a significant association between age and angle of the AEI. The AEI was significantly lower in the older group.

**Table 1:** Comparison of AEI between the Eichner groups.

ANOVA			Post Hoc (LSD)		
Eichner groups	AEI (Mean ± SD)	*p*	Eichner groups		*p*
A	41.36 (7.70)		A	B	0.059
B	38.45 (9.30)	**0.005**	A	C	**0.002**
C	35.68 (8.37)		B	C	0.181

*Note:* Bold values show that AEI was significantly lower in group C than in groups A and B (*p* < 0.05).

**Table 2:** *t*‐Test to compare the AEI between two age groups.

Age group (years)	Mean AEI ± SD	*p*
< 45	42.22 (7.71)	< 0.001
> 45	36.01 (8.20)	

## 4 Discussion

The articular eminence is a small bone in front of the glenoid fossa. Its slope varies among people and changes with chewing force (Chiang et al. 2015; Modgi et al. 2020). TMD is a frequent source of orofacial pain that is not caused by dental issues or infections. Several factors can lead to the signs and symptoms of TMD, with occlusion being a widely debated factor (Paknahad et al. 2023). Identifying the underlying cause of TMD is essential for initiating the appropriate treatment (Trivedi et al. 2022).

This study evaluated the association between the AEI and tooth loss in TMD patients. The results showed a significant association between the Eichner Index and the AEI. This means that the AEI was lower in Eichner's C group than in Eichner's A group. This finding is in agreement with Csadó et al. (2012), who used panoramic images to demonstrate that the AEI decreases with age and tooth loss. Therefore, patients who have lost all their teeth have a much lower articular eminence than those who have at least one occlusal support. Another study showed that the loss of occlusal support affects the AEI more than aging does (Oruba et al. 2020). Another study by Al‐Nakib et al. analyzed the CT images of 117 patients and found that the AEI decreases significantly with tooth loss (Abdul‐Nabi and Al‐Nakib 2015). Our results align with the concept that the TMJ undergoes continuous morphological adaptation throughout life, largely influenced by dental function. As noted in recent CBCT research, the loss of posterior teeth disrupts the equilibrium of the stomatognathic system, leading to bone resorption at the articular eminence. This process results in a flattening of the articular slope and a subsequent decrease in AEI values, suggesting that tooth loss is a primary driver of these anatomical changes (Vîrlan et al. 2024). Our result is consistent with the findings of RauStia et al. (1998) and Modgi et al. (2020). Edentulous patients experience a greater decrease in AEI due to reduced chewing and the types of food they consume. Tooth loss affects TMJ structure by decreasing chewing function, occlusal force, and bone density. It also alters the vertical dimension of occlusion, leading to abnormal loading and changes in TMJ anatomy. These factors can result in a flatter articular eminence (Oruba et al. 2020; Abdul‐Nabi and Al‐Nakib 2015). In addition, Erzurumlu et al. and Indriastuti et al. using OPG, along with Arikan et al. and Vîrlan et al. using CBCT, reported a statistically significant reduction in AEI in edentulous patients compared to those with dentition (Unal Erzurumlu and Celenk 2020; Arıkan et al. 2022; Indriastuti et al. 2019; Vîrlan et al. 2024). When the posterior dentition is lost, the biomechanical balance of the masticatory system is compromised, leading to compensatory anatomical changes within the joint structure (Vîrlan et al. 2021). Some studies, however, find no association between the AEI and tooth loss (Chiang et al. 2015; Suhail et al. 2018; Zabarović et al. 2000; Verma et al. 2020; Chairunnisa and Harahap 2022; Jasinevicius et al. 2006b). These differences in findings may be influenced by factors such as the challenges in measurement due to overlapping components in conventional images and variations in sample sizes.

This study evaluated how age affects the AEI. The patients were divided into two groups based on their age: those who were 45 years old or younger, and those who were older than 45 years. We found a significant association between age and AEI. Sümbüllü et al. (2012) also found a similar result using CBCT images of 93 patients. They reported that the AEI was highest between 21 and 30 years old, and then declined in healthy patients after 31 years old. This may be due to changes in function as people age, leading to alterations in bone structure and a progressively flatter articular eminence over time (Modgi et al. 2020).

This study agrees with previous research in concluding that gender does not affect changes in AEI (Suhail et al. 2018; Sümbüllü et al. 2012). A few studies have compared the AEI between men and women, finding that men generally have a steeper slope than women. However, this difference may be attributed to variations in sample sizes (Chiang et al. 2015; Zabarović et al. 2000). Other studies have used various imaging methods to evaluate the articular eminence. These methods include conventional types (Chiang et al. 2015; Jyothi et al. 2020; Modgi et al. 2020; Chairunnisa and Harahap 2022), as well as CBCT (Sümbüllü et al. 2012), CT (Abdul‐Nabi and Al‐Nakib 2015), and MRI (Ozkan et al. 2012). CBCT has replaced CT for assessing the TMJ due to its ability to accurately measure angles and distances without overlapping structures, as well as its reduced radiation exposure and lower costs (Hintze et al. 2007). Therefore, CBCT images were used in this study.

The current study has several limitations. Firstly, the sample size for each group was relatively small, and there was a gender imbalance, with nearly twice as many females as males. Gender could affect the anatomic changes in the TMJ. Secondly, patient inclusion was based on clinical signs and symptoms recorded in archival data rather than the standardized RDC/TMD or DC/TMD criteria, which may introduce some heterogeneity to the sample. Additionally, other factors such as general health, nutrition, body mass index (BMI), race, duration since tooth loss, and types of prosthetic restorations could also affect the flattening of the AEI. However, these variables were not examined due to the cross‐sectional nature of the study, which only allows for establishing an association between tooth loss and AEI flattening. Furthermore, we could not evaluate disc position using CBCT. While Serindere et al. found that disc displacement is related to eminence morphology (shape), they reported no significant relationship with inclination (angle) (Serindere and Aktuna Belgin 2021). Also, MRI analysis by Nascimento Falcão et al. confirmed a significant correlation between alterations in the articular disc and the morphology of the eminence (Nascimento Falcão et al. 2017). Therefore, the lack of soft tissue assessment remains a limitation. Finally, the study lacked a healthy control group. Since all participants had TMD, it is possible that TMD‐related pathologies (such as inflammation or degenerative remodeling) influenced the AEI values independently of tooth loss.

## 5 Conclusion

Tooth loss can affect the shape of the articular eminence, with patients who have more missing teeth exhibiting a significantly flatter AEI. To prevent morphological changes and potential future problems in the TMJ, it is essential for patients to receive early prosthetic treatments following tooth extraction.

## Author Contributions

Maryam Paknahad was responsible for conceptualization, supervision, project administration, funding acquisition, and writing the original draft. Maryam Paknahad and Leila Haghighi contributed to the methodology. Leila Haghighi performed the formal analysis and contributed to the investigation and visualization. Sonia Dokohaki contributed to the investigation and was responsible for data curation. All authors participated in reviewing and editing the manuscript and approved the final version.

## Funding

The authors received no specific funding for this work.

## Conflicts of Interest

The authors declare no conflicts of interest.

## Data Availability

The data that support the findings of this study are available from the corresponding author, Maryam Paknahad, upon reasonable request.
